# MicroRNA-21 Regulates hTERT via PTEN in Hypertrophic Scar Fibroblasts

**DOI:** 10.1371/journal.pone.0097114

**Published:** 2014-05-09

**Authors:** Hua-Yu Zhu, Chao Li, Wen-Dong Bai, Lin-Lin Su, Jia-Qi Liu, Yan Li, Ji-Hong Shi, Wei-Xia Cai, Xiao-Zhi Bai, Yan-Hui Jia, Bin Zhao, Xue Wu, Jun Li, Da-Hai Hu

**Affiliations:** 1 Department of Burns and Cutaneous Surgery, Xijing Hospital, Fourth Military Medical University, Xi'an, Shaanxi, People's Republic of China; 2 Department of Immunology, Fourth Military Medical University, Xi'an, Shaanxi, People's Republic of China; National Institutes of Health, United States of America

## Abstract

**Background:**

As an important oncogenic miRNA, microRNA-21 (miR-21) is associated with various malignant diseases. However, the precise biological function of miR-21 and its molecular mechanism in hypertrophic scar fibroblast cells has not been fully elucidated.

**Methodology/Principal Findings:**

Quantitative Real-Time PCR (qRT-PCR) analysis revealed significant upregulation of miR-21 in hypertrophic scar fibroblast cells compared with that in normal skin fibroblast cells. The effects of miR-21 were then assessed in MTT and apoptosis assays through in vitro transfection with a miR-21 mimic or inhibitor. Next, PTEN (phosphatase and tensin homologue deleted on chromosome ten) was identified as a target gene of miR-21 in hypertrophic scar fibroblast cells. Furthermore, Western-blot and qRT-PCR analyses revealed that miR-21 increased the expression of human telomerase reverse transcriptase (hTERT) via the PTEN/PI3K/AKT pathway. Introduction of PTEN cDNA led to a remarkable depletion of hTERT and PI3K/AKT at the protein level as well as inhibition of miR-21-induced proliferation. In addition, Western-blot and qRT-PCR analyses confirmed that hTERT was the downstream target of PTEN. Finally, miR-21 and PTEN RNA expression levels in hypertrophic scar tissue samples were examined. Immunohistochemistry assays revealed an inverse correlation between PTEN and hTERT levels in high miR-21 RNA expressing-hypertrophic scar tissues.

**Conclusions/Significance:**

These data indicate that miR-21 regulates hTERT expression via the PTEN/PI3K/AKT signaling pathway by directly targeting PTEN, therefore controlling hypertrophic scar fibroblast cell growth. MiR-21 may be a potential novel molecular target for the treatment of hypertrophic scarring.

## Introduction

Hypertrophic scarring (HS), which is a fibroproliferative disorder caused by abnormal wound healing after skin injury, is characterized by excessive deposition of extracellular matrix and invasive growth of fibroblasts [1]. Although this is a non-malignant disorder, hypertrophic scar fibroblasts (HSFBs) exhibit malignant features. These include excessive deposition and alterations in collagen morphology, excessive proliferation and apoptosis resistance; however, the molecular mechanism underlying HS is not yet fully understood [2]. Previous studies have shown that PTEN (phosphatase and tensin homologue deleted on chromosome ten), functions as a tumor suppressor [3]. Decreased expression of PTEN often results in activation of AKT (pAKT), which is positively correlated with tumor progression [4]. Furthermore, augmentation of PTEN inhibits cancer cell growth, proliferation, survival, and migration [5]. The loss of PTEN function due to deletion, mutation, methylation, or decreased expression has been identified in human cancers [6], [7], [8] and some fibrotic diseases [9], [10]. Abnormal activation of the PI3K/AKT pathway may lead to various diseases including hypertrophic scarring [11]. Indeed, activation of the phosphatidylinositol-3-kinase (PI3K)/AKT pathway promotes dermal fibroblast accumulation [12]. It has been reported that PTEN mediates negative regulation of the PI3K/AKT pathway [13], [14], with some studies showing that PTEN loss enhances PI3K/AKT activation [15]. PTEN is also a key regulator of apoptosis [16]. However, the mechanism of the initial PI3K/AKT activation in HSFBs remains unclear.

Increasing evidence implicates miR-21 as an “oncomir” in tumorigenesis, where it is found to be upregulated in the majority of analyzed cancers, including breast cancer, colorectal cancer, gastric cancer, hepatocellular carcinomas, nasopharyngeal carcinoma, esophageal adenocarcinoma and glioblastoma [17]–[25]. Recent studies have revealed that overexpression of miR-21 can increase cell proliferation, migration, invasion, and metastasis in a variety of cancer cell lines [26]–[30]. Full understanding of the biological functions and molecular mechanisms of the oncomir may provide significant advances in the diagnosis and therapeutic strategies of disease [31], [32]. Previous study has shown that miR-21 downregulates PTEN in a variety of experimental models [33], although miR-21 overexpression has not been shown to induce the loss of PTEN in HS fibroblasts.

In our present study, we demonstrated that miR-21 induced proliferation and inhibited apoptosis in HSFBs. This effect was accompanied by decreased expression of human telomerase reverse transcriptase (hTERT) mediated via the PTEN/PI3K/AKT signal pathway. In addition, we showed that miR-21 mediated direct negative regulation of PTEN by binding to its 3′-UTR leading to inhibition of PTEN translation and activation of the AKT pathway. Moreover, the genes downstream of hTERT, pAKT and PI3K were upregulated by miR-21. This effect was abolished by restoration of PTEN expression. Finally, we observed that miR-21 was upregulated in human HS tissue samples with an inverse correlation between PTEN and hTERT expression observed in these samples. These results suggest that modulation of the mechanism responsible for miR-21 expression in HSFBs could be used as a critical therapeutic strategy for hypertrophic scar intervention and warrants further investigation.

## Materials and Methods

### Tissue samples

Hypertrophic scar (HS) and paired normal skin (NS) tissues were obtained from 16 patients, who were admitted to the Department of Burns and Cutaneous Surgery of Xijing Hospital from May 2009 to June 2013; diagnosis was confirmed by routine pathological examination. Before surgery, all patients were informed of the purpose and procedure of this study and agreed to donate excess tissue. Written informed consent was obtained from all participants involved in this study. All the protocols were approved by the Ethics Committee of Xijing Hospital affiliated to Fourth Military Medical University (China). The collected skin samples were divided into three portions; one was preserved in 4% paraformaldehyde solution for histopathological study, the second was soaked in liquid nitrogen for the preparation of total RNA and total protein lysates, while the third was used for the isolation and culture of fibroblasts.

### Cell culture

Cultures of 15 HSFBs and normal skin fibroblasts (NSFBs) (paired) were established as described previously [34]. All cells were maintained in a humidified incubator at 37°C in an atmosphere containing 5% CO_2_. Fibroblasts obtained at the third to the fifth passages were used in all experiments in this study unless otherwise indicated.

### Transfection of miR-21 mimic and inhibitor

The FAM modified 2′-OMe-oligonucleotides were chemically synthesized and purified by high-performance liquid chromatography (GenePharma, Shanghai, China). The 2′-O-me-miR-21 mimic was composed of RNA duplexes with the following sequence: 5′-UAGCUUAUCAGACUGAUGUUGA-3′;

The sequences of 2′ -O-me-miR-21 inhibitor and 2′ -O-me-scramble oligonucleotides were as follows: 5′-UCAACAUCAGUCUGAUAAGCUA-3′; and 5′-CAGUACUUUUGUGUAGUACAA-3′; All the oligonucleotides were 2′-OMe modified. Transfection with RNA oligonucleotides was established as described previously [35]. Briefly, cells were transfected using Lipofectamine2000 (Invitrogen, CA, USA) at a final concentration of 50 nM. At 24 h post-transfection, the culture medium was changed. After 48 h, cells were harvested for analysis. All transfections were performed in triplicate.

### Quantitative real-time PCR analysis

RNA was extracted from cells using TRIzol Reagent (Invitrogen, CA, USA). Cellular RNA (2 mg) was used for cDNA synthesis. For miR-21 qRT-PCR, total RNA was reverse-transcribed with a miRNA-specific primer using the miScript Reverse Transcription kit (Qiagen, Hilden, Germany). For the detection of the PTEN and hTERT mRNA levels, we employed qRT-PCR using the following primers: PTEN (forward primer: 5′-TGGATTCGACTTAGACTTGACCT-3′; reverse primer: 5′-GGTGGGTTATGGTCTTCAAAAGG-3′; hTERT (forward primer: 5′- AAATGCGGCCCCTGTTTCT-3′; reverse primer: 5′-CAGTGCGTCTTGAGGAGCA-3′. We employed GAPDH mRNA levels as an internal control using the following primers: forward 5′-TCACCAGGGCTGCTTTTAAC-3′; reverse 5′-GACAAGCTTCCCGTTCTCAG-3′. All the miRNA primers (hsa-miR-21; sn-RNU6B) were obtained from AUGCT (Beijing, China) and the reactions were run in triplicate. Relative expression levels of miRNA or mRNA were analyzed using the Bio-Rad C1000 Thermal Cycler (Bio-Rad, CA, USA).

### DNA constructs

The PTEN open reading frame region sequence was ligated into the pcDNA 3.1 vector (pcDNA-PTEN-orf, Genesil, Wuhan, China). The siRNAs were designed to target 19 nt sequences of hTERT mRNA and the empty vector as a control. The hTERT siRNA targeting sequence was as follow: 5′-TCAGACAGCACTYGAAGAG -3′. Paired deoxyribonucleotide oligonucleotides encoding the siRNAs were synthesized, annealed, and cloned into the *Bgl*I and *Hind*III sites of the pSUPER vector (Oligoengine, WA, USA). Stable transfections were performed according to standard protocols as recommended by the manufacturers.

### Luciferase assay

The 3′ UTR of PTEN fragments containing the putative binding site for miR-21 and 3′ UTR-mutation, were modified as previously described [36]. The fragment was then inserted into the 3′-end of the firefly luciferase gene of the dual-luciferase miRNA target expression vector luciferase reporter vector (pGL3) (Promega, WI, USA). HSFBs were added to 48-well plates in duplicate and harvested 24 h later for luciferase assays performed using a kit (Promega, WI, USA). One microgram of pGL3-PTEN-3′-UTR construct was co-transfected with 1 µg of a Renilla luciferase expression construct pRL-TK. Luciferase assays were performed 48 h after transfection using the dual-luciferase reporter assay system. Firefly luciferase activity was normalized to Renilla luciferase expression for each sample.

### Western blotting

Western blotting was performed as previously described [37] using the following antibodies: anti-PTEN mouse monoclonal (ab130224, Abcam, MA,USA), anti-hTERT rabbit polyclonal (ab130224, Abcam, MA, USA), anti-p-AKT rabbit polyclonal (ab81283, Abcam, MA, USA), anti-PI3K mouse polyclonal (ab86714, Abcam, MA, USA) and anti-β-actin mouse monoclonal (A5441, Sigma-Aldrich, MO, USA). Signals were observed using an Odyssey Infrared Imaging System (LI-COR Biosciences, NE, USA).

### Immunocytochemistry

Immunocytochemistry was performed as previously described [34]. Paraffin-embedded hypertrophic scar tissue and normal skin sample slides were immunostained with PTEN and hTERT-specific antibodies (as described for Western blotting) after deparaffinization and hydration. Immunocytochemistry for tissues was performed using the DouSP™ double-staining kit (KIT-9999, Maixin, Fujian, China). Positive signals for the rabbit monoclonal antibody (hTERT) signals appear red. Positive signals for the mouse monoclonal antibody (PTEN) signals appear black.

### Cell proliferation assays and Apoptosis assay

The MTT cell proliferation and apoptosis assay were performed as previously described, [29], [35].

### Statistical analysis

All data are expressed as the mean ±SD of at least three separate experiments. The differences in miR-21 and PTEN expression between hypertrophic scar tissue and normal skin samples were assessed by the Paired-Student's *t*-test; *p*<0.05 was considered statistically significant. Data were analyzed with the PRISM software, version 4 (GraphPad Software, CA, USA)

## Results

### MiR-21 modulates hypertrophic scar fibroblast cell growth and is accompanied by hTERT downregulation

**Figure 1:**
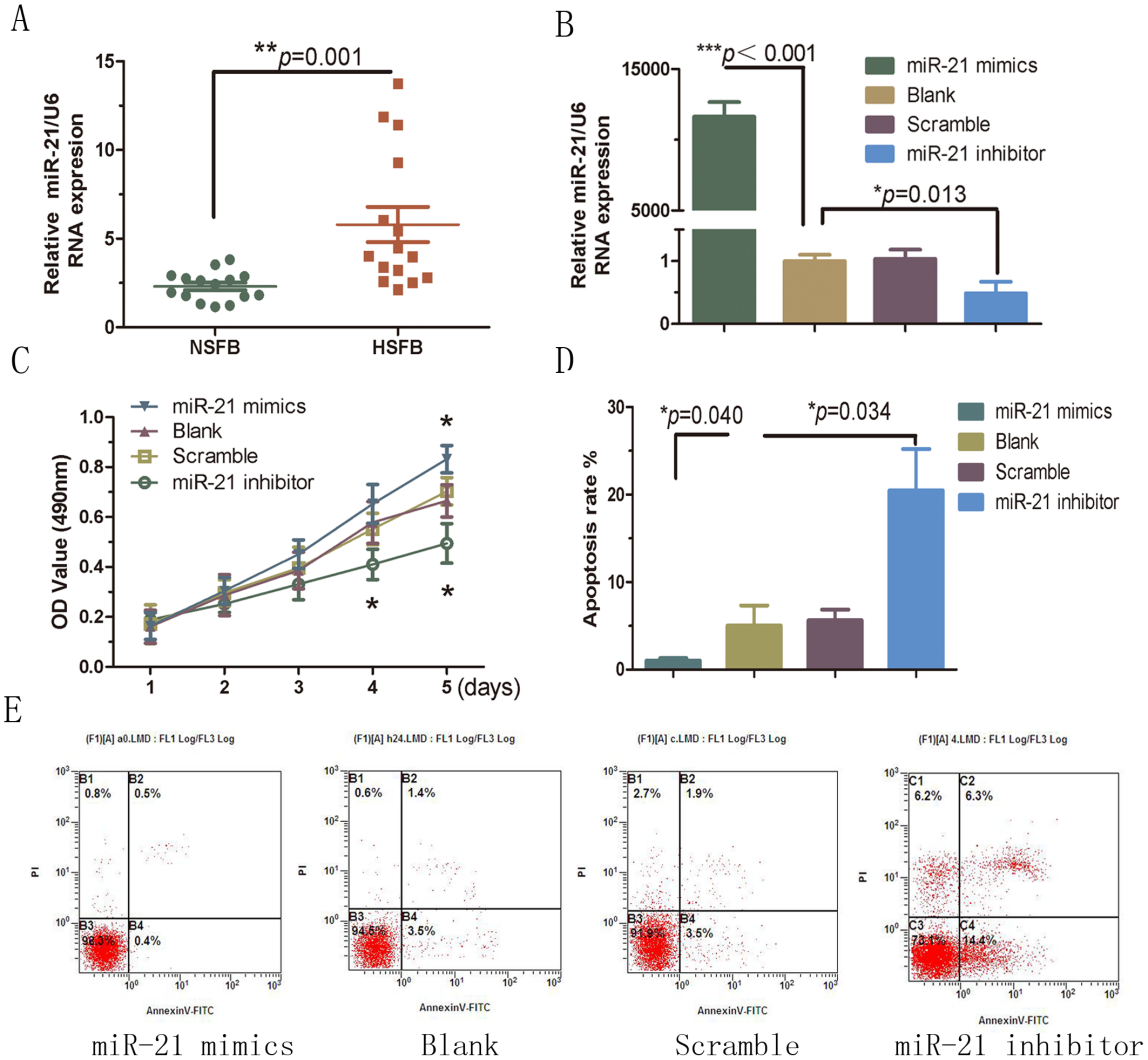
MiR-21 modulates cell growth via hTERT in HSFBs. A, the expression of miR-21 was analyzed by qRT-PCR and normalized to U6 in 15 pairs of NSFBs and HSFBs. The 15 paired HSFB/NSFB samples were cultured from 15 individuals. B, HSFBs were transfected with miR-21 mimic, miR-21 inhibitor or a scrambled oligonucleotide. Non-transfected cells (blank) were used as controls. At 24 h post-transfection, miR-21 expression levels in transfected HSFB and NSFB were evaluated by qRT-PCR analysis. C, MTT assays were performed on all four groups at 24 h intervals for 5 days. D, E, The four groups were transfected for 48 h. Apoptosis was measured by flow cytometric analysis of Annexin V and propidium iodide staining. Blank cells were used as control.

The specific regulation of miR-21 expression in NSFBs and HSFBs (15 samples of each type) was investigated by qRT-PCR analysis. As shown in Figure 1A, miR-21 was significantly overexpressed in HSFBs compared with NSFBs (*p* = 0.001). Aberrant miR-21 expression has also been demonstrated to be associated with a variety of cancers, affecting cells proliferation and invasion [19], [38], [39].

HSFBs were transfected with the miR-21 mimic or inhibitor to evaluate the potential effect of miR-21 in HS. At 24 h post-transfection, miR-21 expression was evaluated by qRT-PCR. The results showed that miR-21 mimics increased the expression of miR-21 (approximately 12,000-fold; *p*<0.001), while the miR-21 inhibitor decreased the expression (approximately 50-fold; *p* = 0.013) compared with the blank group (Figure 1B). Cell viability was measured in MTT assays. As shown in Figure 1C, ectopic expression of miR-21 increased the growth of HSBFs significantly compared to the blank cells (*p*<0.05). MiR-21 inhibitor-treated cells exhibited a significant decrease in proliferation (*p*<0.05). Apoptosis assays revealed significantly more apoptotic HSBFs after transfection with the miR-21 inhibitor compared with the blank groups (*p* = 0.034) (Figure 1D, 1E), while relatively fewer apoptotic cells were detected in the miR-21-mimic-treated group (*p* = 0.040) (Figure 1D, 1E). These results suggested that miR-21 has an impact on cell proliferation and apoptosis, thereby regulating HSFBs growth.

### The PTEN gene is a direct target of miR-21 in HSBFs

**Figure 2:**
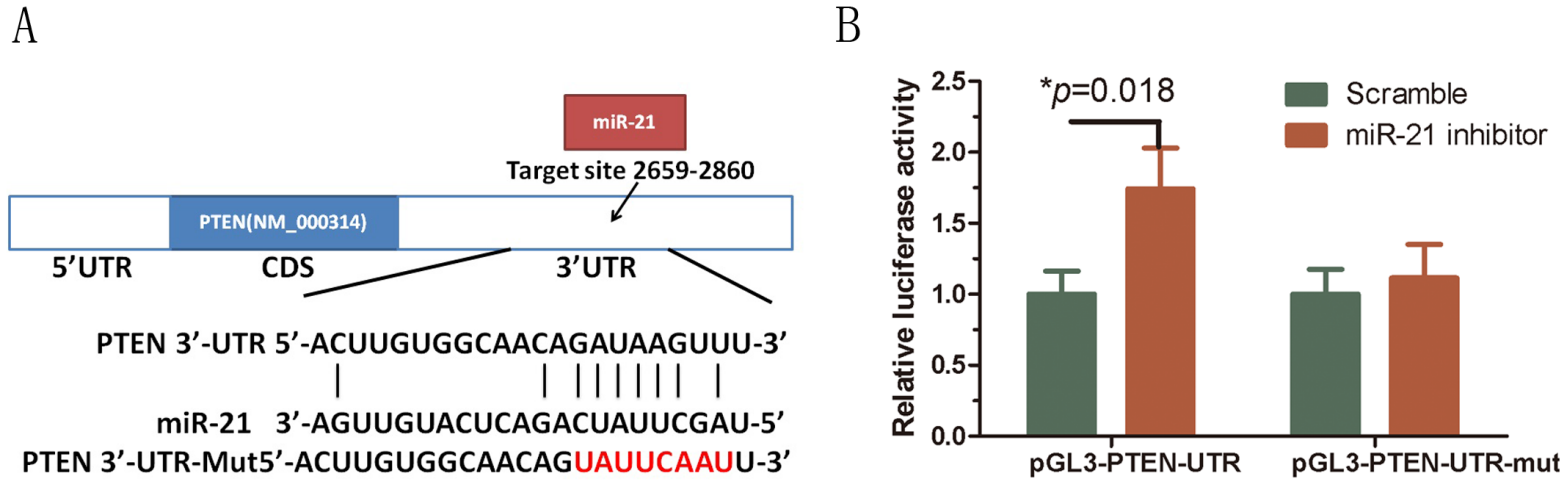
The 3′-UTR of PTEN is a target for miR-21. A, Predicted miR-21 binding sites within the 3′-UTR of PTEN mRNA. The arrows display the mutational nucleotides. B, The wt or mutant reporter plasmid was cotransfected into HSBFs with miR-21 inhibitor or negative control (NC). The normalized luciferase activity in the control group was set as relative luciferase activity. Luciferase activity of pGL3-PTEN was increased significantly by miR-21 inhibitor. However, luciferase activity of pGL3-PTEN-mut was not affected by the miR-21 inhibitor. All data are representative of three independent experiments.

MiRNAs post-transcriptionally silence specific genes via binding to the target mRNAs. We predicted potential targets using the computer-aided algorithms in Targetscan and miRbase Targets. PTEN, a widely expressed tumor suppressor, was identified as a potential miR-21 target gene. The 3′-UTR of PTEN containing the potential miR-21 binding site was cloned for use in a firefly luciferase reporter assay (Figure 2A). The PTEN-UTR or PTEN-UTR-mutant(mut) reporter plasmids were cotransfected into HSFBs along with the miR-21 inhibitor or scramble control [27]. Compared with the scramble control, the miR-21 inhibitor increased the relative luciferase activity significantly when cotransfected with the PTEN-UTR reporter plasmid (*p* = 0.018). However, the mutant reporter plasmid abolished the miR-21 inhibitor-mediated increase in luciferase activity (Figure 2B, *p* = 0.536). These findings suggest that miR-21 suppresses PTEN by direct binding to the 3′-UTR of PTEN.

### MiR-21 regulates PTEN/PI3K/AKT and hTERT

**Figure 3:**
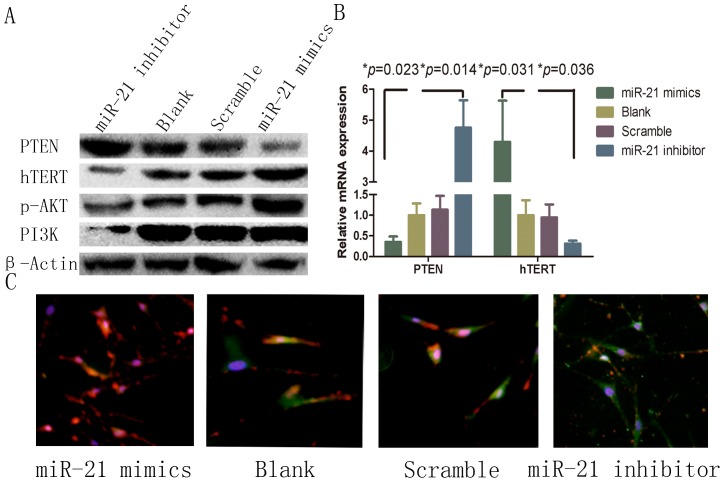
Involvement of the PTEN/PI3K/AKT signaling pathway and hTERT in miR-21 mediated effects. A, Protein expression after transfection of HSFBs. B, PTEN and hTERT mRNA expression after transfection of HSFBs. C, Indirect immunofluorescence of PTEN (green) and hTERT (red) after transfection of HSFBs.

We investigated the effects of miR-21 on gene expression and signaling pathways in HSFBs. In accordance with the results of the luciferase reporter assay results, transient transfection of HFSBs with the miR-21 mimic resulted in a significant reduction in PTEN expression at both the protein (Figure 3A and Figure S1) and mRNA (Figure 3B, *p* = 0.023) levels, while the miR-21 inhibitor-mediated upregulation of PTEN. The PI3K/AKT pathway is an important downstream target of PTEN and PTEN expression results in reduced levels of phosphorylated AKT [35]. Therefore, we investigated the levels of this protein in parallel. As shown in Figure 3A, the expression of phosphorylated AKT and PI3K were decreased by the miR-21 inhibitor. In contrast, transfection of HSFBs with the miR-21 mimic had the opposite effect on phosphorylated AKT and PI3K expression levels. Interestingly, we observed that hTERT, an important gene related to proliferation and apoptosis, was modulated positively by miR-21 at both the protein (Figure 3A and Figure S1) and mRNA levels (Figure 3B, *p* = 0.036). Indirect immunofluorescence staining of PTEN (green) and hTERT (red) displayed inverse levels in miR-21 mimic and miR-21 inhibitor-treated groups. Thus, we demonstrated that PI3K/AKT signaling was altered by targeting PTEN and that PI3K/AKT signaling is moderated by miR-21 at both the mRNA and protein levels.

### Overexpression of PTEN inhibits miR-21-induced proliferation in HSFBs

**Figure 4:**
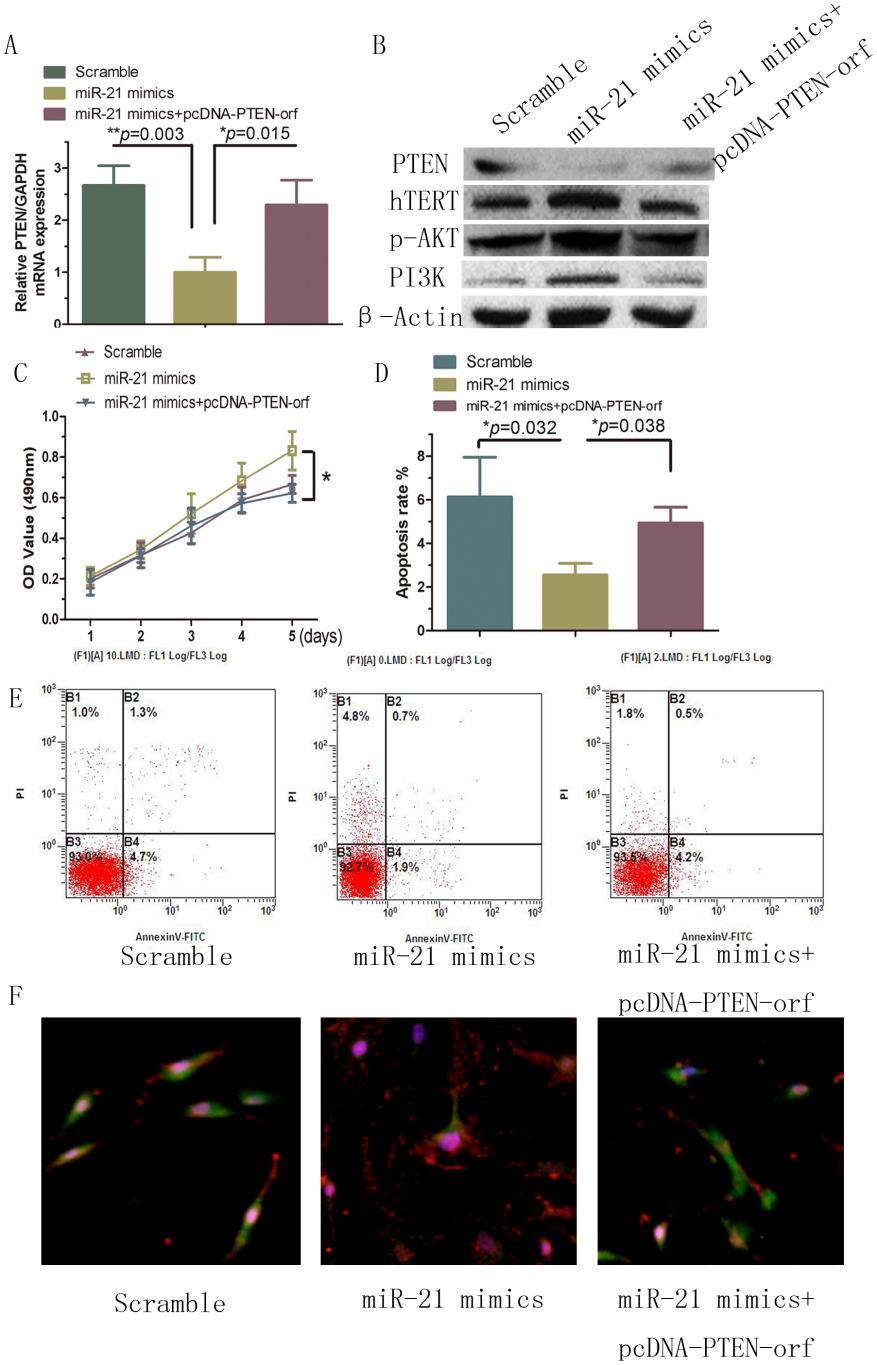
PTEN inhibits miR-21-induced proliferation in HSFBs. A, PTEN mRNA expression; B, signaling pathway protein expression; C, proliferation; D, E, apoptosis were rescued. F, results were confirmed by indirect immunofluorescence staining of PTEN (green) and hTERT (red).

We speculated that if PTEN is a functional target of miR-21 in HSFBs, then the reintroduction of PTEN into cells expressing high levels of miR-21 should antagonize the impact of miR-21 on cell proliferation and apoptosis. To test this hypothesis, we transfected exogenous PTEN into miR-21 mimic-transfected HSBFs. As shown in Figures 4A, Figure 4B and Figure S2, the mRNA (*p* = 0.036) and protein expression levels of PTEN in miR-21-overexpressing cells significantly increased after eukaryotic expression vector-mediated delivery of PTEN. Furthermore, the P13K/AKT signaling pathway and hTERT, which were activated by the miR-21 mimic, were inhibited by PTEN overexpression. Ectopic overexpression of PTEN significantly counteracted the effects on proliferation (Figure 4C) and apoptosis (Figure 4D and 4E) induced by miR-21 in HSBFs. Using indirect immunofluorescence staining (Figure 4F), we confirmed ectopic PTEN (green) re-expression in HSBFs as well as reduced hTERT (red) expression in the miR-21 inhibitor group. Taken together, these data indicate that reintroduction of PTEN abrogates miR-21-induced P13K/AKT protein reduction. At the same time, it also abrogates miR-21-induced effects on cell proliferation and apoptosis, suggesting that PTEN is a functional mediator of the effects of miR-21 in HSFBs. Thus, we speculated that miR-21 regulates hTERT expression via the PTEN/P13K/AKT signal pathway to modulate proliferation and apoptosis in HSFBs.

### Knockdown of hTERT restores the growth-inhibitory and apoptosis-promoting effects of miR-21 in HSFBs

**Figure 5:**
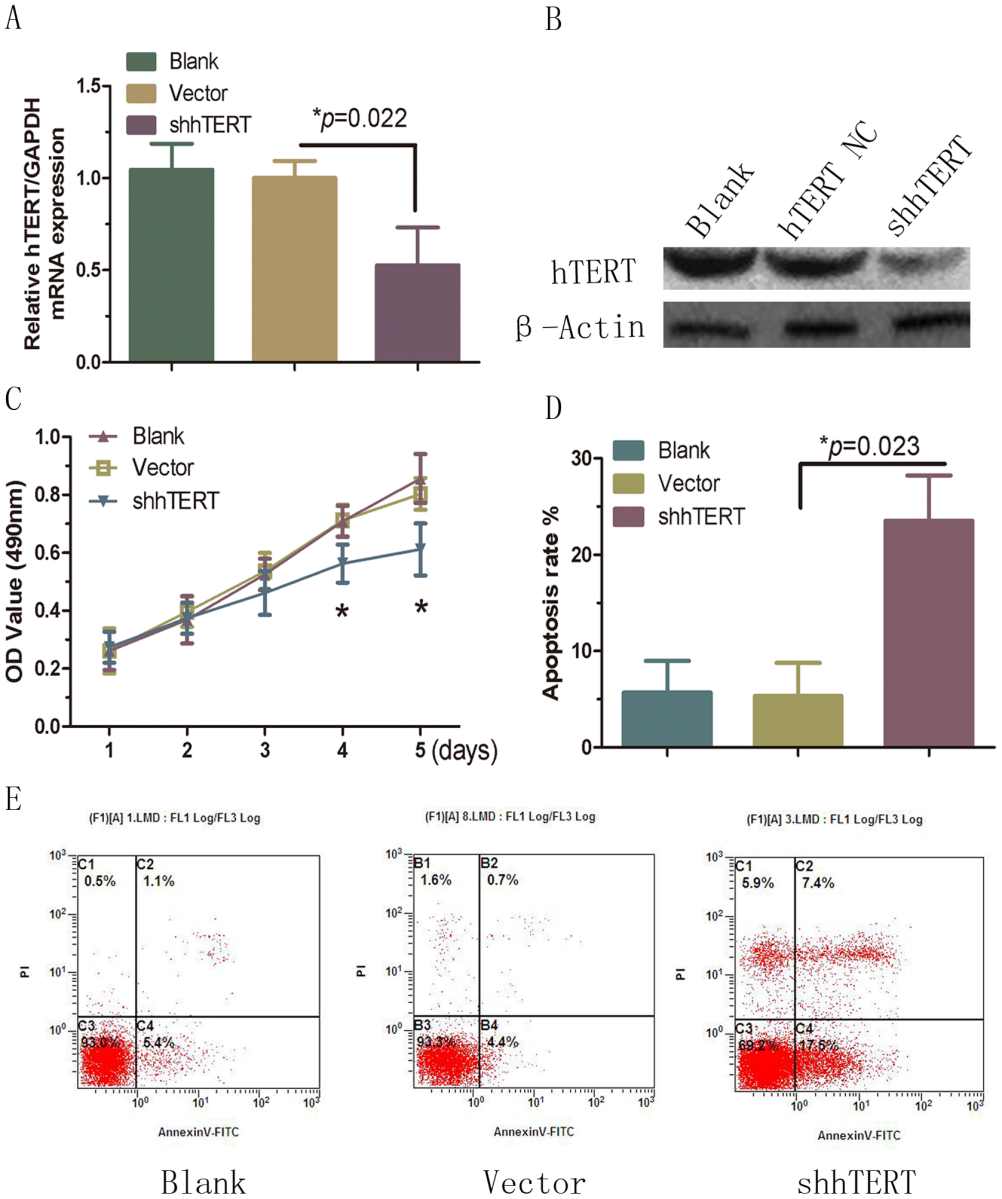
RNAi-mediated hTERT knockdown inhibits HSFBs growth and promotes apoptosis. A, hTERT mRNA expression; B, hTERT protein expression; C, proliferation; D, E, apoptosis were downregulated following RNAi-mediated hTERT knockdown.

Previous studies have indicated that the hTERT overexpression correlates closely with drug sensitivity and tumor growth in carcinoma-associated fibroblasts; however, the mechanism of hTERT activity in HSFBs has not been characterized [40]. To determine whether the biological effect of hTERT in HSFBs is consistent with the proposed functions of miR-21 and PTEN/P13K/AKT signaling, we established cell lines that expressed low levels of hTERT using hTERT-specific RNAi method (Figure 5A and 5B). MTT analysis indicated that hTERT knockdown reduced HSFBs proliferation and increased apoptosis (Figure 5C, 5D and 5E). This was consistent with the effects observed in HSFBs transfected with miR-21, which further confirmed that the downregulation of hTERT is essential for miR-21-induced and PTEN/P13K/AKT-mediated cell proliferation.

### MiR-21, PTEN, hTERT expression in skin tissue samples

**Figure 6:**
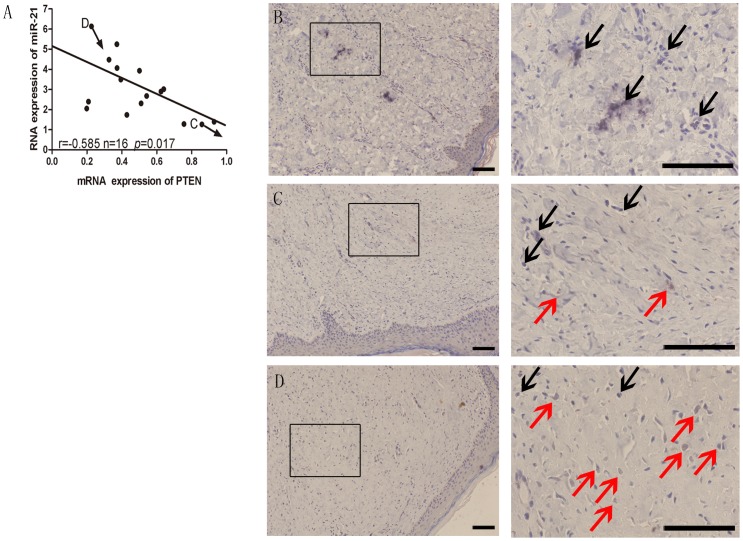
Correlation between miR-21, PTEN and hTERT. A, Inverse correlation of miR-21 and PTEN expression of in HS tissues. B, Immunohistochemical staining of NS tissue; PTEN-positive (black) cells in which miR-21 is expressed at low levels; C, hTERT-positive cells (red) were slightly more prevalent than PTEN-positive cells in HS tissue that express miR-21 at moderate to high levels; D, the number of hTERT-positive cells was extremely high in the high-expressing miR-21 but low-expressing PTEN HS tissue.

It has previously been reported that miR-21 is frequently overexpressed in several types of cancer, including skin tumors [41]. In light of our observation that overexpression of miR-21 led to the downregulation of PTEN mRNA in HSBFs, we postulated an inverse correlation between miR-21 expression and PTEN mRNA expression in HS tissues. To validate this hypothesis, the PTEN mRNA and mature miR-21 expression levels were analyzed in 16 human hypertrophic scar tissue samples by qRT-PCR. As shown in Figure 6A, the expression levels of endogenous miR-21 correlated negatively with the expression levels of PTEN mRNA (Pearson's correlation coefficient  = −0.585; *p* = 0.017). These data provide further evidence of a functional link between miR-21 and PTEN in HS tissues.

Having demonstrated an inverse correlation between miR-21 and PTEN at the RNA level, we investigated PTEN and hTERT expression in normal skin and hypertrophic scar tissues. As shown in Figure 6B, PTEN-positive cells (black) were distributed throughout normal skin, in which miR-21 is expressed at low levels. In contrast, in HS tissues that express miR-21 at moderate to high levels, hTERT-positive cells (red) were slightly more prevalent than PTEN-positive cells (Figure 6C). In particular, the number of hTERT-positive cells was extremely high in the high-expressing miR-21 but low-expressing PTEN HS tissues (Figure 6D). Immunohistochemical double-staining analysis revealed an inverse correlation in the levels of PTEN and hTERT expression in hypertrophic scar tissues expressing high levels of miR-21, thus confirming the in vitro data.

## Discussion

Hypertrophic scarring is one of the most common skin disorders, occurring in 30% to 72% of patients after thermal injury and trauma [42]. HS is cosmetically disfiguring and can cause functional problems that often recur despite surgical attempts to remove or improve the scars. Thus, there is an urgent need to develop novel therapeutic approaches by targeting the molecules that are altered in this dismal disorder. However, the precise mechanisms of hypertrophic scar formation at the molecular and gene expression levels remain to be elucidated. Recently, emerging evidence has demonstrated an important role of miRNAs in scar pathogenesis. MiR-21 has been validated as an oncomir in colorectal, breast, pancreatic, hepatocellular, and gastric cancers [17], [19], [27], [43], [44]. Furthermore, functional studies have documented the potent pro-tumorigenic activity of miR-21 both in vitro and in vivo [45]. However, few studies have described the expression and functions of miR-21 in hypertrophic scarring. It is commonly accepted that HS is the result of high rates of cell proliferation and low rates of apoptosis [46]; therefore, we hypothesized that miR-21 may function as a pro-proliferation and anti-apoptosis factor in HSFBs. In this study, we showed that miR-21 regulated hTERT expression via the PTEN/PI3K/AKT signal pathway, with subsequently moderation of HSFB growth. These findings provide new insights into the mechanism underlying HS formation and therapeutic strategies for this disorder.

The PI3K/AKT pathway is known to be a major cell survival pathway and activation enhances cell survival by stimulating cell proliferation and inhibiting apoptosis [47]. PTEN is a tumor-suppressing dual phosphatase that antagonizes the function of PI3K and negatively regulates AKT activity. Loss of PTEN is thought to be involved in HS pathogenesis and its downregulation can induce PTEN/AKT pathway activation [48]. In addition, Bhalala et al. revealed that miR-21 regulates astrocytic hypertrophy and glial scar progression after spinal cord injury [49].

In this study, we demonstrated that miR-21 regulated PTEN expression by directly targeting the PTEN 3′-UTR. We also demonstrated that transfection of HSFBs with a miR-21 mimic decreased PTEN expression, while inhibition of miR-21 enhanced PTEN expression. This suggested that HSFB proliferation was promoted by AKT activation and that this effect was mediated by decreased PTEN expression resulting from overexpression of miR-21. These observations confirm that PTEN is a functional downstream mediator of miR-21 in HSFBs.

Telomerase activity, which is essential for the maintenance of replicating tumor cell integrity and the establishment of immortality, is required for the survival of the large majority of tumor cells [50]. It is widely accepted that telomerase dysfunction is associated with both abnormal fibrogenesis and carcinogenesis [51]. Here we showed that hTERT siRNA inhibited cell proliferation and induced cell apoptosis in HSFBs, which is consistent with the effects of hTERT-loss reported in other cell models [52]. Until now, telomerase expression in HS and HSFBs has not been well-documented. In this study, hTERT expression was increased at both the mRNA and protein levels in HSFBs compared with NSFBs. At the same time, high levels of hTERT detected by immunohistochemistry showed a correlation with low levels of PTEN. hTERT, the catalytic unit in the telomerase complex, is controlled by many factors, one of which is PTEN, which acts as a negative regulator [53]. Here, we also revealed that stable transfection with PTEN decreased hTERT mRNA and protein expression. The exact functional significance of the miR-21/PTEN/hTERT axis in hypertrophic scar formation remains to be clarified. However, in this study, we showed that reduction in miR-21 expression repressed hTERT expression via the PTEN/PI3K/AKT signaling pathway, leading to the subsequent inhibition of HSFB growth.

## Supporting Information

Figure S1 **Protein expression of the PTEN/PI3K/AKT signaling pathway and hTERT in miR-21-mimic-treated HSFBs.** Values shown are the mean±SD for each group from three independent experiments. All the band intensities are normalized to the blank controls.
(TIF)

Figure S2 **Protein expression of miR-21-mimic and pcDNA-PTEN-orf transfection in HSFBs.** Values shown are the mean±SD for each group from three independent experiments. All the band intensities are normalized to the scramble groups.
(TIF)
