# Supplementary figures and images for: MicroRNA-21 Regulates hTERT via PTEN in Hypertrophic Scar Fibroblasts
Source: PLoS One. 2014 May 9;9(5):e97114. doi: 10.1371/journal.pone.0097114 (PMC4016251; doi:10.1371/journal.pone.0097114)

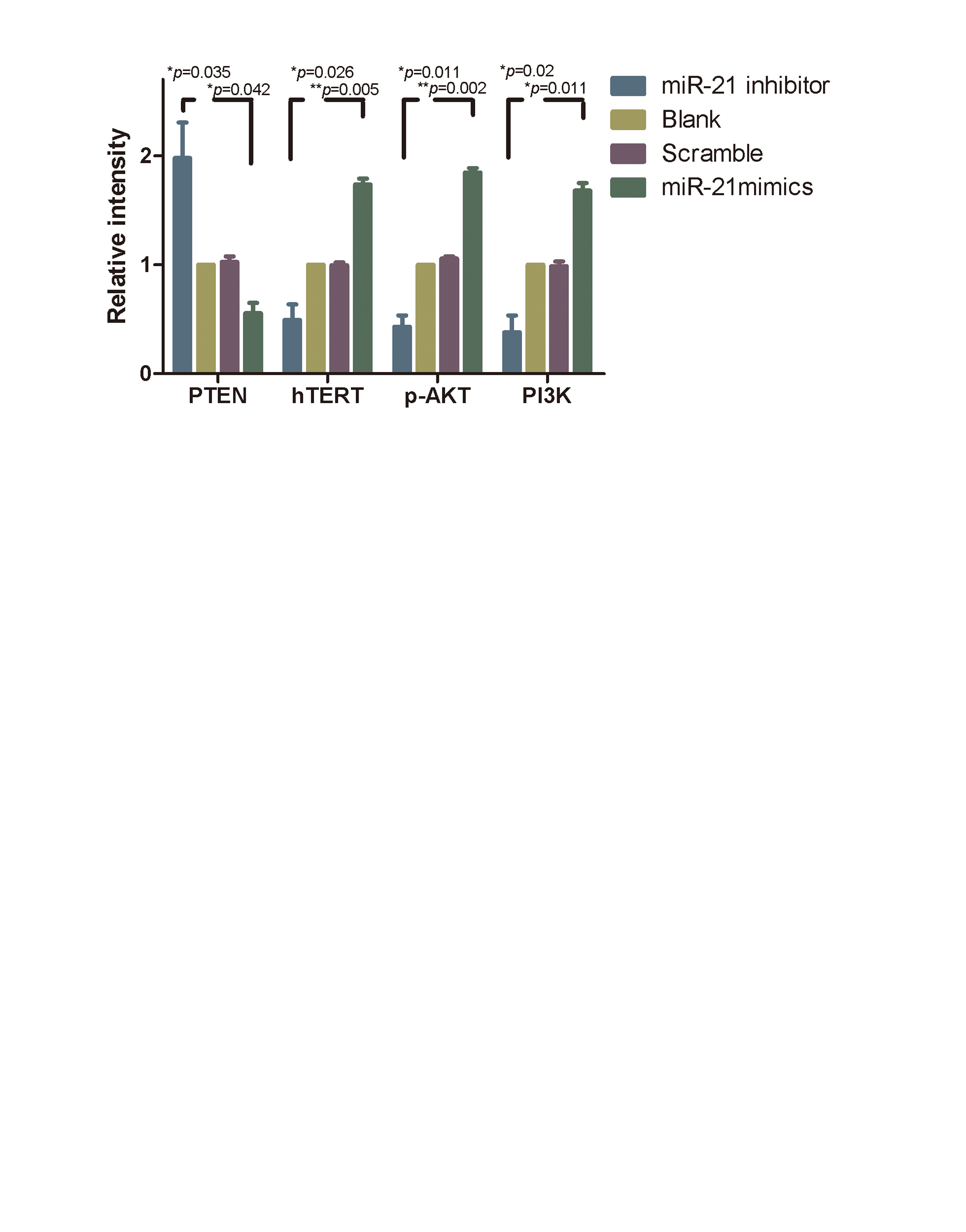

Supplement: Figure S1 — Protein expression of the PTEN/PI3K/AKT signaling pathway and hTERT in miR-21-mimic-treated HSFBs. Values shown are the mean±SD for each group from three independent experiments. All the band intensities are normalized to the blank controls. (TIF) [file pone.0097114.s001.tif]

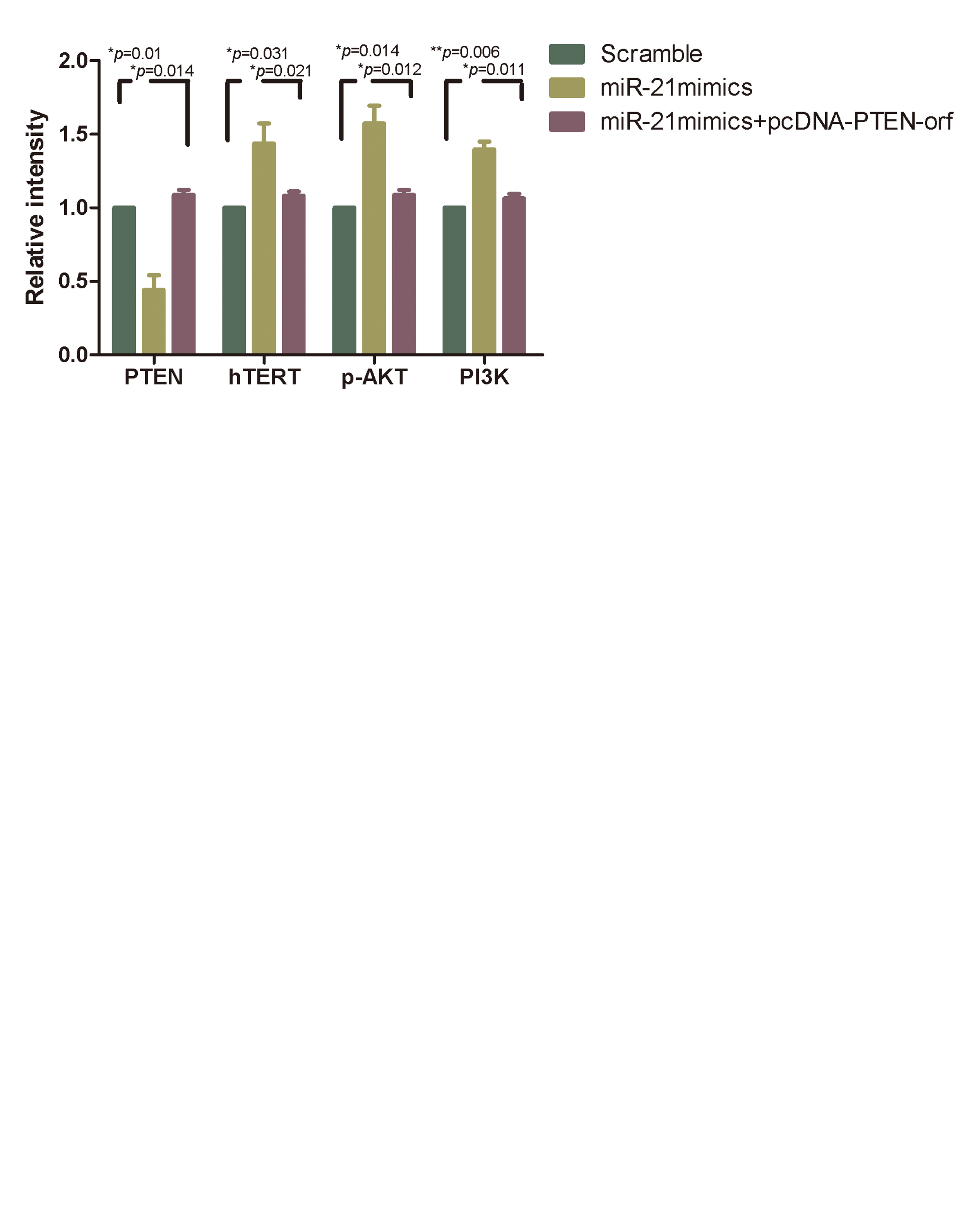

Supplement: Figure S2 — Protein expression of miR-21-mimic and pcDNA-PTEN-orf transfection in HSFBs. Values shown are the mean±SD for each group from three independent experiments. All the band intensities are normalized to the scramble groups. (TIF) [file pone.0097114.s002.tif]
